# Traceability of “Tuscan PGI” Extra Virgin Olive Oils by ^1^H NMR Metabolic Profiles Collection and Analysis

**DOI:** 10.3390/metabo8040060

**Published:** 2018-09-30

**Authors:** Chiara Roberta Girelli, Laura Del Coco, Samanta Zelasco, Amelia Salimonti, Francesca Luisa Conforti, Andrea Biagianti, Daniele Barbini, Francesco Paolo Fanizzi

**Affiliations:** 1 Department of Biological and Environmental Sciences and Technologies, University of Salento, Prov.le Lecce-Monteroni, 73100 Lecce, Italy; chiara.girelli@unisalento.it (C.R.G.); laura.delcoco@unisalento.it (L.D.C.); 2 Council for Agricultural Research and Economics–Research Centre for Olive, Citrus and Tree Fruit C. da Rocchi, 87036 Rende (CS), Italy; samanta.zelasco@crea.gov.it (S.Z.); amelia.salimonti@crea.gov.it (A.S.); 3 CNR-Institute of Neurological Sciences, Località Burga, Piano Lago, 87050 Mangone (CS), Italy; francescaluisa.conforti@cnr.it; 4 Certified Origins Italia *srl*, Località il Madonnino, 58100 Grosseto, Italy; andrea.biagianti@oleificioolma.it (A.B.); daniele.barbini@oleificioolma.it (D.B.)

**Keywords:** chemometrics, protected designation of origin (PDO), protected geographical indication (PGI), extra virgin olive oil (EVOO), Nuclear Magnetic Resonance (NMR), genetic analysis

## Abstract

According to Coldiretti, Italy still continues to hold the European Quality record in extra virgin olive oils with origin designation and protected geographical indication (PDO and PGI). To date, 46 Italian brands are recognized by the European Union: 42 PDO and 4 PGI (Tuscan PGI, Calabria PGI; Tuscia PGI and PGI Sicily). Specific regulations, introduced for these quality marks, include the designation of both the geographical areas and the plant varieties contributing to the composition of the olive oil. However, the PDO and PGI assessment procedures are currently based essentially on farmer declarations. Tuscan PGI extra virgin olive oil is one of the best known Italian trademarks around the world. Tuscan PGI varietal platform is rather wide including 31 specific olive cultivars which should account for at least 95% of the product. On the other hand, while the characteristics of other popular Italian extra virgin olive oils (EVOOs) cultivars from specific geographical areas have been extensively studied (such as those of Coratina based blends from Apulia), little is still known about Tuscan PGI EVOO constituents. In this work, we performed, for the first time, a large-scale analysis of Tuscan PGI monocultivar olive oils by ^1^H NMR spectroscopy and multivariate statistical analyses (MVA). After genetic characterization of 217 leaf samples from 24 selected geographical areas, distributed all over the Tuscany, a number of 202 micro-milled oil samples including 10 PGI cultivars, was studied. The results of the present work confirmed the need of monocultivar genetically certified EVOO samples for the construction of ^1^H-NMR-metabolic profiles databases suitable for cultivar and/or geographical origin assessment. Such specific PGI EVOOs databases could be profitably used to justify the high added value of the product and the sustainability of the related supply chain.

## 1. Introduction

Originally known as *oleaster*, the olive tree appeared more than 6000 years B.C. for the first time in Asia Minor and successively diffused in the countries of the Mediterranean basin [1]. To date, olive groves spread still continue all around the world and extra virgin olive oil (EVOO), in particular, remains undoubtedly the most important production of Mediterranean countries, due to its nutraceutical, antioxidant and other well-known health properties [2]. Among other vegetable oils, EVOO is a premium price product in the national and international market, leading to the risk of adulteration and mislabelling [3]. Thus, EVOO authenticity and traceability are important for consumer health and commercial purposes. Severe standards on olive oil production, origin and labelling have been established by European Community (EC) Council of Regulation [4]. In particular, traceability was defined as “the ability to trace and follow a food, feed, food-producing animal or substance intended to be, or expected to be incorporated into a food or feed, through all stages of production, processing and distribution” [5]. In order to improve and protect the high-quality products from a particular origin, the European Regulation (EC, 1992, EC, 2006), established rules on the Protection of Designations of Origin (PDO) and Protected Geographical Indications (PGI) of agricultural products and foodstuff [6,7]. In 2012, the EU Regulation 1151/2012 introduced new guidelines on quality system for agricultural product including PDO, PGI and TSG (Traditional Specialty Guaranteed) schemes. For their well-defined geographic origin, some olive cultivars are recognized as higher quality agricultural products and included in the PDO/PGI labelling [8,9,10]. For Regulation purposes (EU 510/06, Article 2), both the PDO and PGI indicate “the name of a region, a specific place or, in exceptional cases, a country, used to describe an agricultural product or a foodstuff: originating in that region, specific place or country” [6]. They differ in the quality definition, being for PDO: “the quality or characteristics of which are essentially or exclusively due to a particular geographical environment with its inherent natural and human factors” and for PGI “specific quality, reputation or other characteristics attributable to that geographical origin”.

Moreover, PGI definition is assigned to agricultural and food products whose at least one stage of the production process must be performed within the defined geographical area. On the contrary, for PDO, the entire production cycle must be carried out in a specific territory [6,10]. Thus, PGI labelling focuses on quality, reputation and specific characteristics related to the geographical origin [10]. To date, world olive oil production is concentrated (98%) in the Mediterranean basin and, in particular, Spain (45%) and Italy (15%) [11]. Together with Spain, Italy accounts for almost all world exports (60% Spain and 20% Italy). Moreover, Italy still continues to hold the European quality record in EVOOs with 40% of protected designation origin and protected geographical indication (PDO and PGI) [12]. In particular, 46 Italian brands are recognized by the European Union, distinguished in 42 PDO and 4 PGI (Tuscan PGI, Calabria PGI, Tuscia PGI and PGI Sicily). Tuscan PGI extra virgin olive oil is one of the best known Italian trademarks around the world, increasingly diffused and commercialized, especially in the U.S.A. market. Tuscan PGI varietal platform is rather wide including 31 specific olive cultivars, which should account for at least 95% of the product [13]. On the other hand, while the characteristics of other popular Italian EVOO cultivars from specific geographical areas have been extensively studied (such as Coratina based blends and “Terra di Bari” PDO EVOOs from Apulia) [14,15,16,17], little is still known about Tuscan PGI EVOO constituents. Combination of environmental aspects with olive cultivars genetic characteristics resulted in the metabolic profile of a specific product. It is largely known, that oil characteristics, such as fatty acid composition, minor and volatile compounds, organoleptic and nutraceutical properties, are strictly related with genetic patrimony. Nevertheless, they also depend on environmental conditions, agronomical practises and local adaptation of the different olive growing [1]. Besides official methods of analysis to be used as reference in defining satisfactory physical and chemical characteristics of EVOOs [18], several alternative methodologies have been also proposed for determining oils profiles. Thanks to their screening potential, several instrumental techniques have been used for this purpose, such as GS-MS, UV-VIS, Raman, NIR, Mass, NMR and others [19]. These latter (Mass and NMR), being the most successfully *high-throughput* among the spectroscopic techniques, have been widely used for food screening and, in particular, for characterization of olive oil authenticity, adulteration and traceability [3,20,21,22,23]. In this work we performed, for the first time, a large-scale analysis of Tuscan PGI monocultivar olive oils by ^1^H NMR spectroscopy and multivariate statistical analyses (MVA). A number of 217 leaf samples were collected from 24 selected geographical areas, distributed all over the Tuscany and at first genetically characterized using a set of 10 microsatellite markers (SSRs). Molecular analysis revealed a ~93% varietal correspondence of oils (202 on 217 accessions) with 10 Tuscan PGI cultivars. Thus, 202 oil samples were obtained by micro-milling from olives collected from genetically certified localized trees. The aim of this work is to verify the possibility of assessing PGI classification by using ^1^H NMR metabolic profiles databases and MVA (multivariate analysis) beside farmers declarations. This work could also provide a contribution to support the extra virgin olive oil based economy of the local region. A scientifically certified quality could validate the high added value of the product, promoting its use by end customers (both in Italy and on the foreign markets) and buttressing the sustainability of the related supply chain.

## 2. Results and Discussion

A preliminary genetic characterisation of the plant material (217 olive leaf samples, Table S1) was successfully performed in order to check the correct cultivar declaration of the samples analysed. Genetic analysis revealed that 157 samples were correctly assigned to the declared cultivars, (Table S2) even if a few cases of intra-cultivar variation was shown (Table S3).

A number of 45 samples declared has been reassigned to Tuscan cultivars (Table S2). It is worth noting that the cultivars declared “Morchiaio” corresponded to the Tuscan variety called “Giogolino” which is not included in the Tuscan PGI specification (Tables S3 and S4). Overall, 202 accessions corresponded to Tuscan PGI varietal platform while 12 varieties were found to be outside of the composition of the Tuscan PGI production disciplinary and 3 accessions were not identified (Table S2). In particular, two unidentified accessions were genetically identical each other for the 10 SSR markers used and they could be to consider known variety (Table S3). The CREA-OFA internal database includes all the Tuscan PGI olive varieties except for two —named “Scarlinese” and “Melaiolo”—making impossible the genetic identification of these cultivars.

The main Tuscan PGI varieties found were Frantoio, Leccino, Moraiolo, Pendolino followed by Maurino, Leccio del Corno while not more than 1 accession of minor varieties such as Mignolo, Mignolo cerretano, Olivastra Seggianese, Rossellino was found. The Tuscan PGI specification includes 31 specific olive cultivars, which should account for at least 95% of the product. In this study, we found in a sample of 217 accessions collected in different geographical areas of Tuscany, a correct PGI varietal composition of about 90% of accessions. All the varieties not belonging to Tuscan PGI specification were discarded.

**Figure 1:**
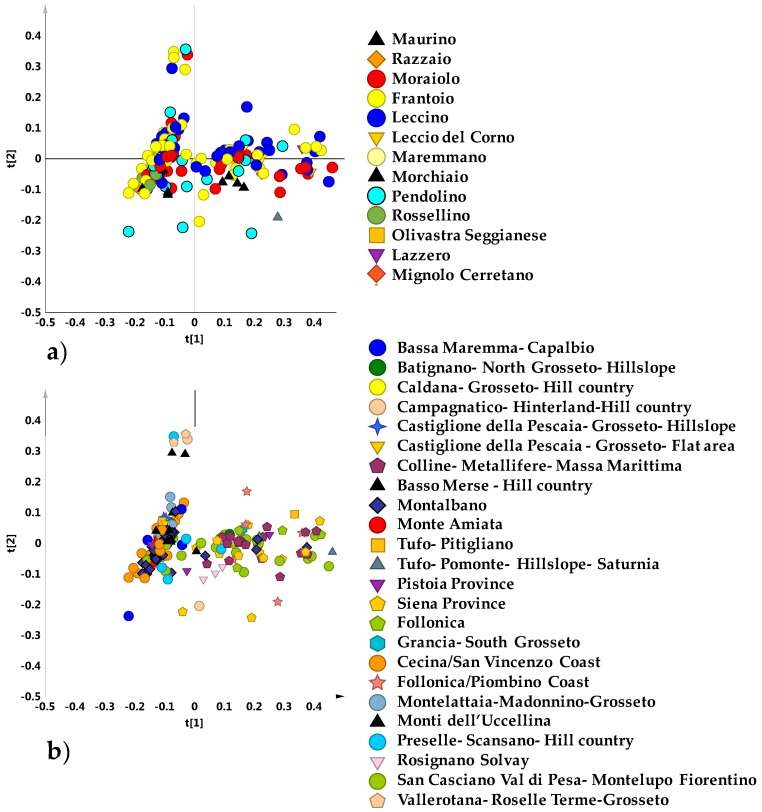
Principal Component Analysis (PCA) t[1]/t[2] scores plot (t[1] and t[2] explain 56.7% and 14.5% of the total variance, respectively) for micro-milled olive oil samples labelled according to declared cultivar (**a**) and geographical area (**b**).

In order to investigate the general trend of data grouping, the whole NMR dataset (obtained from 202 micro-milled olive samples) was studied. An explorative unsupervised PCA analysis of the NMR dataset was first performed. The PCA model obtained with five components gave R^2^X = 0.89 and Q^2^ = 0.77). The PCA t[1]/t[2] scores plot for the model, with samples labelled according to the declared cultivars (Figure 1a) or geographical origin (Figure 1b) showed that no specific clustering among samples could be observed.

**Figure 2:**
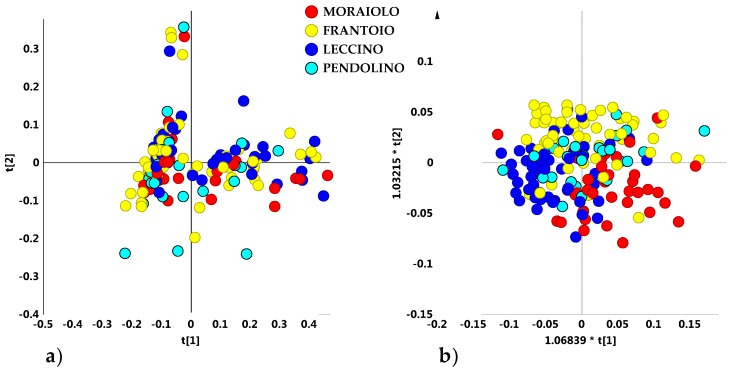
(**a**) PCA t[1]/t[2] (t[1] and t[2] explain 57.3% and 14.6% of the total variance, respectively) and (**b**) OPLS-DA (3 + 3 + 0; R^2^X = 0.91; R^2^Y = 0.24; Q^2^ = 0.13) t[1]/t[2] scores plots for main cultivar micro-milled olive oil samples.

Nevertheless, a rough separation of samples in two main groups, apparently independent on both declared cultivar or geographical origin, was observed, specifically along the first principal component t[1]. In order to identify a possible clustering among samples according to cultivar, an unsupervised analysis (PCA five components give R^2^X = 0.89; Q^2^ = 0.73) was performed, by considering only olive oil samples belonging to the four main cultivars (those present in a representative number for each cultivar and therefore statistically more significant): Moraiolo, Frantoio, Leccino and Pendolino. Also in this case, the PCA scores plot showed a dispersion of the samples without any specific separation among the cultivars. However, the existence of two macro-groups discriminated along the main component was confirmed. A more compact group was found at negative values of PC1 component, distinct from a dispersed macro area, this last at positive values of PC1 (Figure 2a). The supervised OPLS-DA analysis (3 + 3 + 0; R^2^X = 0.91; R^2^Y = 0.24; Q^2^ = 0.13) did not improve the separation among the cultivars and the resulting model lacked in a significant predictive capability (Q^2^ = 0.13) (Figure 2b). 

**Figure 3:**
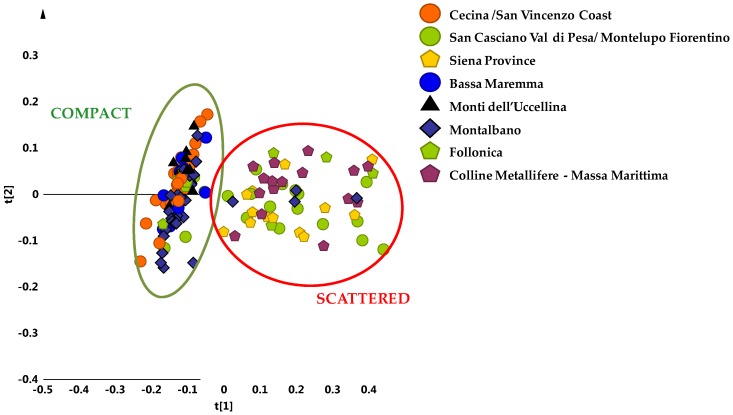
PCA t[1]/t[2] scores plot for micro-milled olive oil samples from the main geographical origin area (t[1] and t[2] explain 70% and 9% of the total variance, respectively). Compact and scattered macro groups were identified by green and red circles respectively.

It should be noted that, a different behaviour was found in the case of four of the main cultivars from Apulia Region (Southern Italy) [16]. A clear discrimination among the most popular olive cultivar of the Apulia region, Coratina and three popular local cultivars used as “sweeteners” in Coratina-based blends (Ogliarola, Cima di Mola and Peranzana) from the Bari and Foggia provinces (Apulia region, Southern Italy) was observed in two different harvesting years [15,24]. With the aim to obtain similar results also in the case of PGI Tuscan oils, the analysis was focused of the main reference areas, which were represented with at least 9 samples per area. Despite the sample classes reduction, the corresponding unsupervised PCA model (5 components, R^2^X = 0.93 and Q^2^ = 0.82) showed again and even more clearly the existence of a sample clustering in two different macro groups, specifically along the first principal component t[1] (Figure 3).

**Figure 4:**
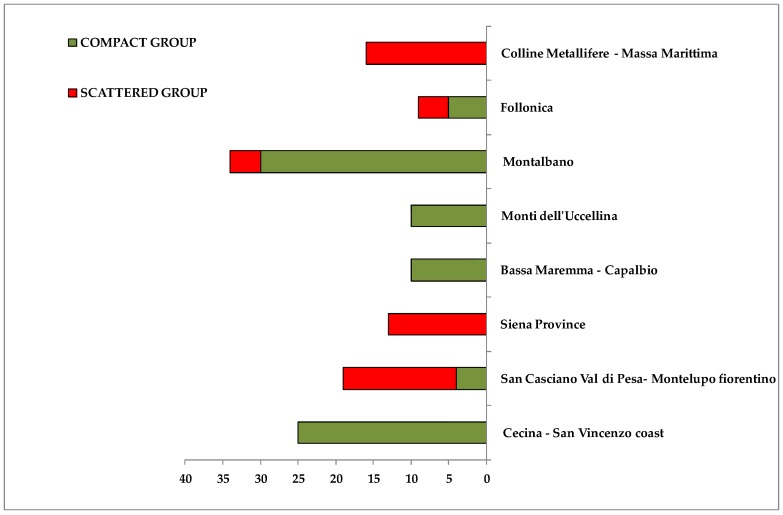
Bars chart representing percentage distribution of samples from each main geographical origin areas into two identified macro groups. Green and red rectangles indicate percentage contribution to the compact and scattered macro-groups respectively. X axis reported the number of samples.

A compact group was observed at negative values of t[1], which was clearly distinct from the other scattered cluster, found at positive values of the same principal component t[1]. In the first group (compact macro area) it was possible to identify some geographical areas of origin for the oil samples: Montalbano (30 samples), Cecina San Vincenzo Coast (25 samples), Bassa Maremma di Capalbio (10 samples), Monti dell’ Uccellina (10 samples), Follonica (5 samples) areas and San Casciano Val di Pesa-Montelupo Fiorentino (4 samples). On the contrary, at positive values of the first component t[1], the scattered cluster consisted of oils from olives collected in San Casciano Val di Pesa/Montelupo Fiorentino (15 samples), Siena province (13 samples) and Colline Metallifere/Massa Marittima (16 samples) areas, Follonica (4 samples) and Montalbano (4 samples). It should be noted that oils from Cecina San Vincenzo Coast, Bassa Maremma di Capalbio and Monti dell’ Uccellina were observed only in the compact macroarea, while samples from Siena province and Colline Metallifere-Massa Marittima were identified only in the scattered area (Figure 4).

**Figure 5:**
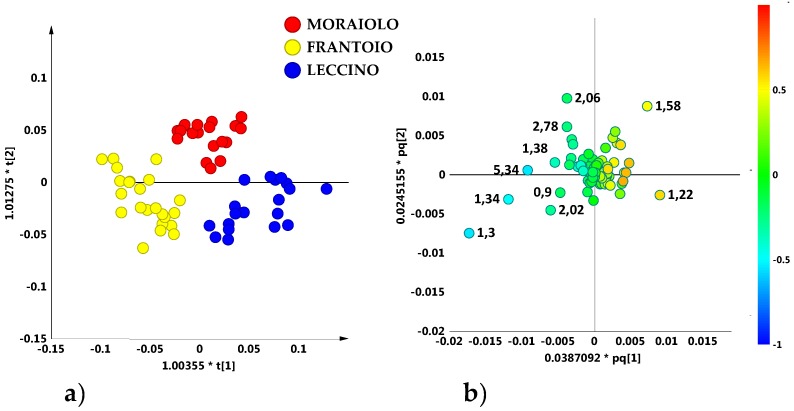
(**a**) OPLS-DA (2 + 4 + 0 components give R^2^X = 0.83, R^2^Y = 0.74, Q^2^ = 0.57) scores plot for main cultivar micro-milled olive oil samples from the observed compact macro group. (**b**) Loading scatter plot for the model indicating the molecular component responsible for the cultivar separation. NMR spectra with detailed assignment of discriminating metabolites are reported as Figure S1 in SI.

Therefore, by considering oil samples from the main reference areas, at least a certain degree of separation among the PGI Tuscan oils on the basis of the geographical origin could be obtained. In order to deeply analyse this samples distribution, unsupervised and supervised analyses were performed again, by considering now separately the identified macro-groups. The OPLS-DA supervised analysis, built with the samples belonging to the compact macro-group and considering the most representative cultivars (Frantoio, Moraiolo, Leccino), gave a good descriptive and predictive model (2 + 4 + 0 components, R^2^X = 0.83, R^2^Y = 0.74 and Q^2^ = 0.57) (Figure 5a) revealing a certain degree of separation among the main representative cultivars. In particular, Leccino and Frantoio samples were clearly distinct from each other along the first predictive component (t[1]), while the Moraiolo class was found along the second component t[2] and located in a central position of the graph, differently from the other two oil groups. Pendolino oil samples were excluded from the model because they were too scattered. The corresponding loading plot for the model allowed to highlight the molecular components responsible for the class separation. In particular, Leccino cultivar was characterized by a high content of saturated fatty acids (1.26 ppm), while a high content of oleic acid (1.30 ppm) characterized the Frantoio class. Finally, the polyunsaturated fatty acids (PUFA) (2.06, 2.78, 1.58 ppm) were responsible for separation of the Moraiolo class (Figure 5b) from the other two classes. 

**Figure 6:**
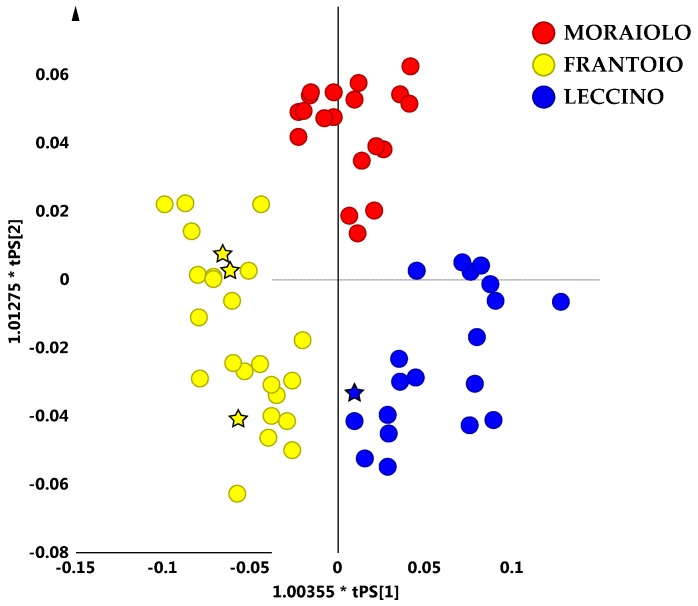
OPLS-DA (2 + 4 + 0 components give R^2^X = 0.83, R^2^Y = 0.74, Q^2^ = 0.57) predicted scores plot for main PGI cultivar micro-milled olive oil from the observed compact macro-group. The predicted samples are indicated as five points stars coloured as declared cultivar oils.

The OPLS-DA model built using the most representative olive cultivars of the compact macro-group (Leccino, Frantoio and Moraiolo) was also successfully used as a prediction model, in order to assign some test samples (specifically monovarietal oils), supplied by the provider. From a simple visual inspection of the OPLS-DA predicted scores plot, it was possible to correctly assign the oil test samples in the model (Figure 6).

Moreover, this was confirmed by analyzing the confusion matrix for the prediction model (Table 1), in which the correctly classified samples in the prediction set were shown. Therefore, in principle, as already observed for 100% Italian [15] and PDO EVOOs [14] this model could be also profitably used in order to assess blends of the specific cultivars originating from these specific geographical areas.

**Figure 7:**
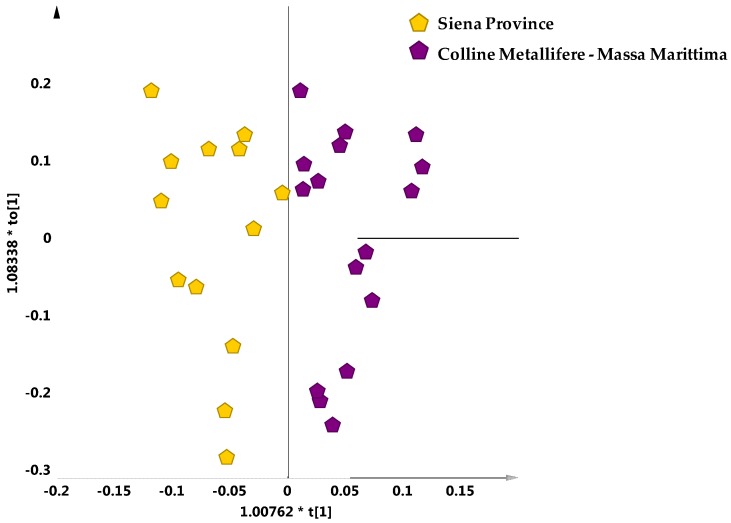
OPLS-DA (1 + 1 + 0 components give R^2^X = 0.74, R^2^Y = 0.75, Q^2^ = 0.65) scores plot for samples from two main referenced geographical areas and exclusively present in the scattered macro group.

The supervised analysis (OPLS-DA) was then applied considering only the samples falling in the scattered macro-group of the PCA scores plot. No specific separation among the main reference cultivars (Leccino, Frantoio and Moraiolo) was observed (data not sown). On the other hand, a clear separation for these oil samples could be observed by OPLS-DA, according to geographical origin. Indeed, looking at the two main geographical areas, exclusively present in the scattered macro group, the Siena province could be clearly differentiated from Colline Metallifere–Massa Marittima samples with good descriptive and predictive capabilities of the statistical model (1 + 1 + 0; R^2^X = 0.74, R^2^Y = 0.75, Q^2^ = 0.65) (Figure 7). This suggests that for the samples of the scattered macro-group of the PCA scores plot the geographical origin, rather than olive cultivars was the most predominant discriminating factor on cluster separation.

**Figure 8:**
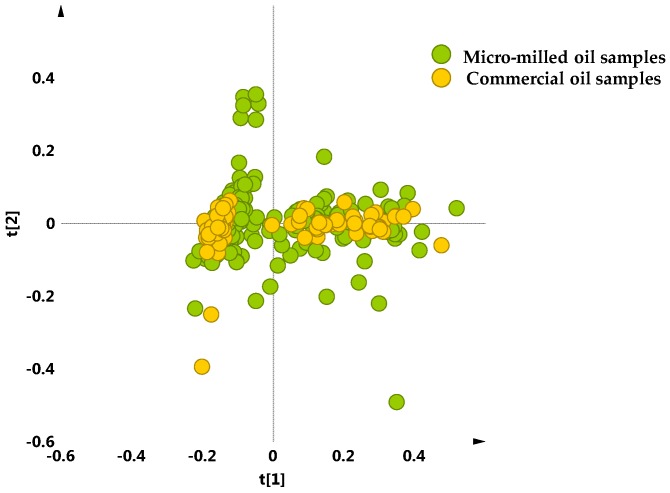
PCA t[1]/t[2] scores plot (t[1] and t[2] explain 59.5% and 13.2% of the total variance, respectively) for micro-milled olive oil samples and commercial bottled protected geographical indication (PGI) EVOOs, supplied by *Certified Origins Italia srl*.

A crosscheck was also performed in order to assess the significance of micro-milled samples used in this study with respect to the commercial ones. The unsupervised analysis (PCA five components give R^2^X = 0.88, Q^2^ = 0.75) carried out on both the oil samples obtained from laboratory micro-milling and the commercial bottled of multi- and monovarietal PGI Tuscan oils did not show any relevant separation among the different olive oil extraction procedures. Actually, as observed for micro milled oil samples, the scores plot which includes the commercial samples, showed the same distribution in two macro-groups, one more compact and one more dispersed (Figure 8). 

**Figure 9:**
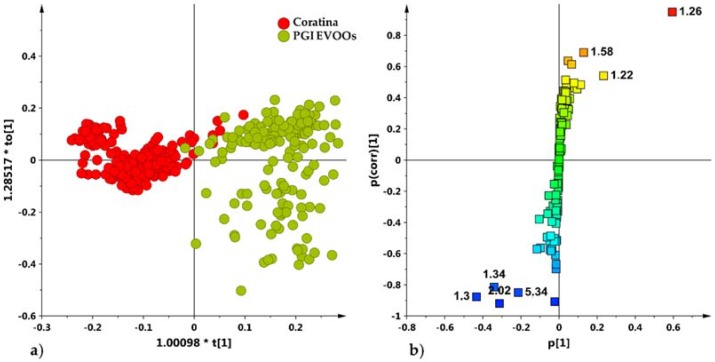
(**a**) OPLS-DA (1 + 1 + 0 components give R^2^X = 0.75, R^2^Y = 0.85, Q^2^ = 0.85) scores plot for Coratina cultivar [16] and micro-milled olive oil samples (**b**) *S*-line plot for the model displaying the predictive loadings coloured according to the correlation scaled loading [p(corr)].

Finally, a comparison among the PGI micro milled oils and the main popular Apulian cultivar (Coratina) [16] was performed. The OPLS-DA supervised analysis (1 + 1 − 0) gave a model with good significance parameters (R^2^X = 0.75, R^2^Y = 0.85, Q^2^ = 0.85) and showed a clear separation between the Tuscan oils and the Apulian Coratina cultivar (Figure 9a). This indicates that, despite being more dispersed and complicated, the Tuscan PGI EVOOs here studied can be clearly distinguished with respect to the popular and easily available Apulian, [12] Coratina oils. The *S*-plot (Figure 9b) for the model identified the molecular component responsible for the separation between the cultivar.

As already known [16], Coratina samples were characterized by a high relative content of monounsaturated (i.e., oleic acid) (loadings at 1.30, 2.02 ppm) and polyunsaturated fatty acids (linoleic and linolenic acids) (loadings at 1.34, 5.34 ppm), while PGI Tuscan EVOOS showed relative high values of saturated fatty acids (1.22 and 1.26 ppm).

## 3. Materials and Methods

### 3.1. Sampling

**Figure 10:**
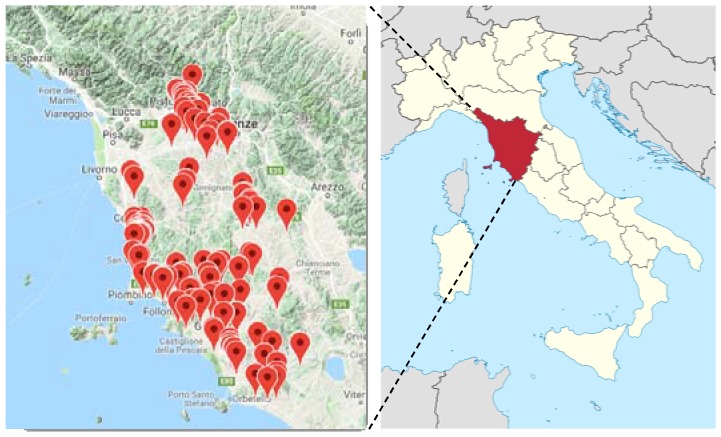
The samples collected from each area are indicated with map markers in the expansion of the Tuscan region (Italy). (from http://www.progettott.info/www/MappaNMR.php; https://en.wikipedia.org/wiki/Tuscany).

A number of 217 samples (for both olives and leaves, Table S1), supplied by *Certified Origins Italia s.r.l*., were collected during the harvesting period 2016–2017, from 24 different georeferenced selected Tuscany areas (Figure 10).

**Figure 11:**
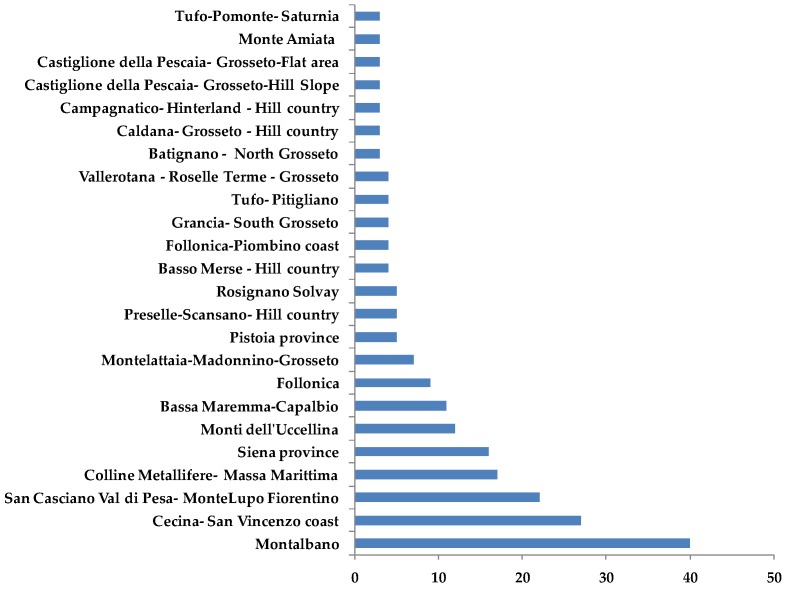
Bars-chart representing samples (supplied by *Certified Origins Italia s.r.l*.) distribution in the geographical reference areas.

Samples came essentially from eight geographical areas with a high level of geomorphologic heterogeneity [28]: Montalbano, Cecina San Vincenzo Coast, San Casciano Val di Pesa-Montelupo Fiorentino, Colline Metallifere-Massa Marittima, Siena Province, Monti dell’ Uccellina, Bassa Maremma-Capalbio and Follonica (Figure 11).

**Figure 12:**
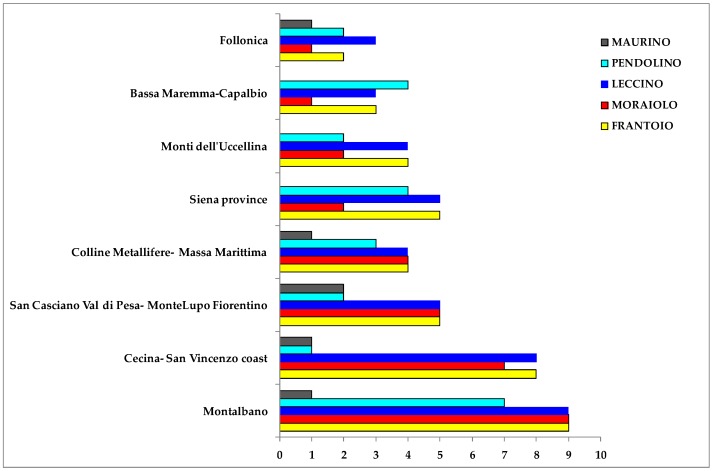
Bars-chart representing samples (supplied by *Certified Origins Italia s.r.l*.) distribution of most representative olive cultivars in the main geographical reference areas.

The most representative declared olive cultivars were Frantoio, Leccino, Moraiolo, Pendolino and Maurino, with 57, 55, 42, 37 and 11 oil samples, respectively. Other minor declared cultivars were Leccio del Corno (4 samples), Rossellino (3 samples), Morchiaio (3 samples), Lazzero, Maremmano, Mignolo cerretano Olivastra seggianese and Razzaio, these last with 1 samples for each cultivar. About 70% of the most representative olive cultivars samples were collected from the main geographical areas (Figure 12).

### 3.2. SSR Analysis and Varietal Identification

Molecular characterization was conducted on 217 olive leaf samples, with a set of 10 microsatellite markers. Sampled leaves were collected from olive plants and immediately placed in a paper envelope with silica gel. Samples were kept in a box for a dehydration process. 5 mg of dried leaf tissue was ground using Tissuelyser II (QIAGEN) and subsequently resuspended in 100 µL of distilled sterile water and then vortexed for 30 s, 1 µL of each leaf sample in distilled sterile water was amplified. PCRs were performed using KAPA3G Plant DNA polymerase (KAPA Biosystems) in a reaction mix with the following composition: 2X KAPA Plant PCR buffer, 100X KAPA Plant PCR Enhancer, 25 mM MgCl_2_, 10 mM of pair of primers. Forward primers were labelled with specific fluorochromes (6-FAM, VIC, PET and NED). Different combinations of three SSR loci were used in multiplex PCR amplification strategy. DCA3-6Fam, DCA5-VIC, DCA8-VIC, DCA11-PET and DCA18-6Fam [29], GAPU71B-6Fam [30], UDO12-NED and UDO15-NED [31], EMO090-6Fam [32] and OLEST23-PET [33] loci were used in this work. KAPA3G Plant DNA polymerase 2U was added in a final volume of 25 µL. The thermal profile, in the Veriti^TM^ thermal cycler (Applied Biosystems), was 10 min at 95 °C and 50 cycles composed of 30 s at 95 °C, 15 s at 55 °C and 30 s at 72 °C with a final elongation at 72 °C for 1 min, as reported by Migliaro et al. [34].

Amplification products were separated on a Genetic Analyzer 3130xl (Applied Biosystems Inc., Foster City, CA, USA). The main authenticated Tuscany cultivars held in CREA-OFA olive tree collection located in Mirto Crosia (CS) were included into the analysis as internal reference to verify the correctness of molecular data. SSR fragments were analyzed by Gene Mapper 3.7 software (Applied Biosystems, Foster City, CA, USA). The obtained data by scoring of SSR profiles were used to calculate a similarity matrix using Dice’s coefficient [35]. The similarity values were utilized to determine the cluster analysis based an unweighted pair group method with arithmetic mean (UPGMA) using PAST software v.2.12.

In order to carry out the varietal identification of the accessions outside of the IGP Tuscan varietal platform, molecular data obtained in this study, were harmonized and compared with those from the internal CREA-OFA standardized database. The harmonization was carried out by shifting of one or more single repeat for each allele in comparison with reference one. For the loci SSR GAPU71b, DCA3, DCA5, DCA18, GAPU71b and EMO090, reference alleles were taken from oleadb database [36], reference alleles for the loci SSR DCA11 and UDO12 came from [37], UDO 15 from [38], while for the locus OLEST 23 were the same used in Reference [39]. 

### 3.3. Olive Oil Extraction

Oils were extracted from olive samples by using a laboratory scale milling method, in a short time, reducing any type of decomposition due to thermal effects. For each sample, olives (20 g) were plunged into liquid N_2_ and ground to obtain a paste with a stainless- steel blender. After storing over night at 4 °C the past was added of 2–4 mL of distilled water and centrifuged. The oil (about 2–4 mL) was collected from the upper phase and stored in amber vials until NMR analysis.

### 3.4. ^1^H NMR Analysis and Data Processing

NMR samples were prepared dissolving ~140 mg of olive oil in CDCl_3_ and adjusting ratio of olive oil: CDCl_3_ to 13.5: 86.5 (% *w*/*w*). This ratio was chosen to give the best trade-off for sensitivity/solution viscosity in spectral acquisition (Bruker Italia, standardized procedure for olive oil) [16]. Next, 600 µL of the prepared mixture were transferred into a 5-mm NMR tube. ^1^H NMR spectra were recorded on a Bruker Avance spectrometer (Bruker, Karlsruhe, Germany) operating at 400.13 MHz, T = 300 K, equipped with a PABBI 5-mm inverse detection probe incorporating a z axis gradient coil. NMR experiments were performed after sample randomization to avoid biasing results due to instrument conditions or operator related differences. The entire process was conducted under full automation for the entire process, after loading individual samples on a Bruker Automatic Sample Changer (BACS-60), interfaced with the IconNMR software (Bruker). In order to optimize NMR conditions, automated tuning and matching, locking and shimming and 90° hard pulse calibration P(90°) were done for each sample using standard Bruker routines ATMA, LOCK, TOPSHIM and PULSECAL. After a 5-min waiting period for temperature equilibration, a standard one-dimensional (^1^H ZG) NMR experiment was performed for each sample. The relaxation delay (RD) and acquisition time (AQ) were set to 4 s and ~3.98 s, respectively, resulting in a total recycle time of ~7.98 s. Free Induction Decays (FIDs) were collected into time domain (TD) = 65,536 (64 k) complex data points by setting: spectral width (SW) = 20.5524 ppm (8223.685 Hz), receiver gain (RG) = 4 and number of scans (NS) = 16, usually used for samples where metabolites are present in high concentrations, as in the case of olive oil analysis [24,40]. NMR data were processed using Topspin 2.1 (Bruker). ^1^H NMR spectra were obtained by the Fourier Transformation (FT) of the FID (Free Induction Decay), applying an exponential multiplication (EM) with a line broadening factor of 0.3 Hz, automatically phased and baseline corrected. Chemical shifts were reported with respect to the TMS (internal standard) signal set at 0 ppm, obtaining good peak alignment.

### 3.5. Multivariate Statistical Analysis

^1^H NMR spectra were processed by segmentation in rectangular fixed (0.04 ppm width) buckets and integration by Amix 3.9.15 (Analysis of Mixture, Bruker BioSpin GmbH, Rheinstetten, Germany) software. Bucketing was performed within 10.00–0.5 ppm region, excluding the residual non-deuterated chloroform signal and its carbon satellites signals (7.6–6.9 ppm). The total sum normalization was applied to minimize small differences due to olive oil concentration and/or experimental conditions among samples [41,42,43]. The Pareto scaling method (performed by dividing the mean-centered data by the square root of the standard deviation) was then applied to the variables. The prior log transformation of the data (Figure S2) did not improve the final outcome of the MVA [44,45,46]. Therefore, no further pre-processing, including noise removal [45] was used. The data table generated by all aligned buckets row reduced spectra was used for multivariate data analysis. Each bucket row represents the entire NMR spectrum, with all the molecules in the sample. Moreover, each bucket in a buckets row reduced spectrum is labelled with the value of the central chemical shift for its specific 0.04 ppm width. The variables used as descriptors for each sample in chemometric analyses are the buckets. Multivariate statistical analysis and graphics were obtained using Simca-P version 14 (Sartorius Stedim Biotech, Umeå, Sweden). PCA (Principal Component Analysis), PLS-DA (data not shown) and OPLS-DA (Partial Least Squares and Orthogonal Partial Least Squares Discriminant Analyses, respectively) were applied to the data [47,48,49]. Principal Component Analysis is at the basis of the multivariate analysis [47] and usually performed to extract and display the systematic variation in a data matrix X formed by rows (the considered observations), in our case the EVOO samples and columns (the variables describing each sample) in our case the buckets from each NMR spectrum. In this work, the PLS-DA method was also performed in order to justify the number of components used in OPLS-DA model [50]. The OPLS-DA analysis is a modification of the usual PLS-DA method which filters out variation that is not directly related to the response and produces models of clearer interpretation, focusing the predictive information in one component, as shown in several recent studies of metabolomics [26,51]. The OPLS-DA is the most recently used technique for the discrimination of samples with different characteristics (such as cultivars and/or geographical origin). The further improvements made by the OPLS-DA in MVA resides in the ability to separate the portion of the variance useful for predictive purposes from the not predictive variance (which is made orthogonal) [52]. OPLS-DA models are useful tools in application of prediction and classification. The related classification list and confusion matrix summarize the probability of belonging to the class models, showing correctness or incorrectness of particular sample classification [53]. In order to evaluate the robustness and predictive ability of the statistical models, a seven-fold cross-validation procedure was performed [54,55,56]. Moreover, the minimal number of required components can be easily defined by the analysis of R^2^ and Q^2^ parameters, which display completely diverging behaviour as the model complexity increases. The R^2^X, R^2^Y and Q^2^, describing the total variation in X, the variation in the response variable Y and the predictive ability of the models, respectively, were calculated [57]. The results were shown by the optimal bidimensional scores plots and relative loadings plots, which were used to identify differences among groups [58].

### 3.6. Chemicals

All chemical reagents for analysis were of analytical grade. CDCl_3_ (99.8 atom %D) and tetramethylsilane, TMS (0.03 *v*/*v* %) were purchased from Armar Chemicals (Döttingen, Switzerland).

## 4. Conclusions

The present study represents the first large-scale analysis of oils obtained from cultivars and geographical areas specific for the production of Tuscan PGI EVOOs. Analyzing the NMR-based metabolomic profiles of both laboratory micro-milled and commercial oil samples, a distribution of the samples in two main macro-groups was observed by PCA analysis. The first one, which includes more samples, results in a more compact cluster, while the second group gives a more dispersed one. The different statistical models built by considering separately these two groups showed a very different samples distribution characteristics. In the first case, (compact macro-group samples) a separation of the main reference cultivars, Frantoio, Moraiolo and Leccino, appeared the most relevant discriminating feature with satisfactory model parameters and good predictive capabilities. On the other hand, the scattered macro-group samples could be reasonably well separated only on the basis of the geographical areas rather than olive cultivars. These results showed, for the first time, the specificity of the Tuscan PGI EVOOs production. The observed high variability of this product depends not only on the numerous PGI allowed local cultivars but also on the high heterogeneity of the pedoclimatic conditions characteristic of the region. This result is in contrast with the characteristics of the EVOOs coming from extensively studied most popular Apulian cultivars and geographical areas [14,15,16,17]. Further studies are required to deeply characterize the Tuscan PGI EVOOs, especially in the scattered macro-group geographical areas such as Siena Province, Colline Metallifere—Massa Marittima area). Nevertheless all the here reported Tuscan PGI EVOOs could be clearly distinguished with respect to the popular and easily available Apulian, [12] Coratina oils. The results of the present work confirmed the need of monocultivar genetically certified EVOO samples for the construction of a ^1^H-NMR-metabolic profiles database suitable for cultivar and/or geographical origin assessment. Such a specific PGI EVOOs database could be profitably used to justify the high added value of the product and the sustainability of the related supply chain.

## Figures and Tables

**Table 1:** Misclassification table for the model. Y predicted value estimates class affiliation and the limit of 0.65 was chosen for the assignment of observations to a specific class. The observations with no Y predicted below 0.65 were not assigned (no class column). Each observation was assigned to the nearest class.

	Members	Correct	Moraiolo	Frantoio	Leccino	No Class (YPred ≤ 0.65)
**Moraiolo**	19	100%	19	0	0	0
**Frantoio**	23	100%	0	23	0	0
**Leccino**	19	100%	0	0	19	0
**No class**	4		0	3	1	0
Total	65	100%	19	26	20	0
Fisher’s probability ^1^	5.5 × 10^−22^					

^1^ Fisher’s exact test is derived from the probability of the particular classification result and all outcomes more extreme than the one observed. All probabilities more extreme than the observed pattern are computed and summed to give the probability of the table occurring by chance [25]. Assignments were performed according to Naïve–Bayes classification (Software WEKA 3.8, University of Waikato New Zealand), [26,27] (Tables S5).

## Supplementary Materials

The following are available online at http://www.mdpi.com/2218-1989/8/4/60/s1, Table S1: List of olive samples from geographical areas within Tuscany region (Italy), Table S2: Molecular characterization results of the declared accessions collected in eight Tuscany areas, Table S3: Molecular profile of declared accessions correctly identified. In bold: allele variant from the reference cultivar, Table S4: Reference molecular profiles of Tuscan PGI declared cultivars in this work, Table S5: Classifier Output from Weka analysis, Figure S1: Representative ^1^H NMR spectra of Moraiolo, Leccino, Frantoio olive oil samples. Figure S2: Data pre-treatment.
