# Supplementary material for: Traceability of “Tuscan PGI” Extra Virgin Olive Oils by 1H NMR Metabolic Profiles Collection and Analysis
Source: Metabolites. 2018 Sep 30;8(4):60. doi: 10.3390/metabo8040060 (PMC6316653; doi:10.3390/metabo8040060)
Supplement: Supplementary file 1 [file metabolites-08-00060-s001.zip › TableS5.docx]

**Table S5**

**Modello 103-OPLS-DA predizione di campioni su moraiolo leccino e frantoio**

WEKA software

https://www.cs.waikato.ac.nz/ml/index.html

=== Run information ===

Scheme: weka.classifiers.bayes.NaiveBayes

Relation: WekaExcel

Instances: 61

Attributes: 4

Ypred_a (moraiolo)

Ypred_b (frantoio)

Ypred_c (leccino)

class

Test mode: evaluate on training data

=== Classifier model (full training set) ===

Naive Bayes Classifier

Class

Attribute a b c

(0.31) (0.38) (0.31)

=======================================

Ypred_a

mean 0.7898 0.0891 0.1066

std. dev. 0.1445 0.2444 0.2293

weight sum 19 23 19

precision 0.0236 0.0236 0.0236

Ypred_b

mean 0.1063 0.8591 0.068

std. dev. 0.1612 0.1252 0.2958

weight sum 19 23 19

precision 0.0269 0.0269 0.0269

Ypred_c

mean 0.1039 0.0529 0.8282

std. dev. 0.1599 0.2514 0.1582

weight sum 19 23 19

precision 0.027 0.027 0.027

=== Evaluation on training set ===

Time taken to test model on training data: 0 seconds

=== Summary ===

Correctly Classified Instances 61 100 %

Incorrectly Classified Instances 0 0 %

Kappa statistic 1

Mean absolute error 0.0014

Root mean squared error 0.0077

Relative absolute error 0.3226 %

Root relative squared error 1.6305 %

Total Number of Instances 61

=== Confusion Matrix ===

a b c <-- classified as

19 0 0 | a = a

0 23 0 | b = b

0 0 19 | c = c

=== Detailed Accuracy By Class ===

TP Rate FP Rate Precision Recall F-Measure MCC ROC Area PRC Area Class

1,000 0,000 1,000 1,000 1,000 1,000 1,000 1,000 a

1,000 0,000 1,000 1,000 1,000 1,000 1,000 1,000 b

1,000 0,000 1,000 1,000 1,000 1,000 1,000 1,000 c

Weighted Avg. 1,000 0,000 1,000 1,000 1,000 1,000 1,000 1,000

=== Evaluation on test set ===

=== Summary ===

Correctly Classified Instances 4 100 %

Incorrectly Classified Instances 0 0 %

Kappa statistic 1

Mean absolute error 0.002

Root mean squared error 0.0049

Relative absolute error 0.4683 %

Root relative squared error 1.0799 %

Total Number of Instances 4

=== Detailed Accuracy By Class ===

TP Rate FP Rate Precision Recall F-Measure MCC ROC Area PRC Area Class

? 0,000 ? ? ? ? ? ? a

1,000 0,000 1,000 1,000 1,000 1,000 1,000 1,000 b

1,000 0,000 1,000 1,000 1,000 1,000 1,000 1,000 c

Weighted Avg. 1,000 0,000 1,000 1,000 1,000 1,000 1,000 1,000

=== Confusion Matrix ===

a b c <-- classified as

0 0 0 | a = a

0 3 0 | b = b

0 0 1 | c = c

the Naïve Bayes classification was implemented by WEKA 3.8 (University of Waikato, New Zealand) <https://www.cs.waikato.ac.nz/ml/index.html>
