# Supplementary material for: Traceability of “Tuscan PGI” Extra Virgin Olive Oils by 1H NMR Metabolic Profiles Collection and Analysis
Source: Metabolites. 2018 Sep 30;8(4):60. doi: 10.3390/metabo8040060 (PMC6316653; doi:10.3390/metabo8040060)
Supplement: Supplementary file 1 [file metabolites-08-00060-s001.zip › Figure S1.docx]

**Figure S1.** Representative ^1^H NMR spectra of Moraiolo, Leccino, Frantoio olive oil samples. The peaks of significant metabolites are indicated with letters (a. 2.74-2.78 ppm, =CH–C*H*_2_–CH=, (PUFA diallilyc groups); b. 2.32-2.26 ppm, –OCO–C*H_2_* (acyl group); c. 2.07-1.96 ppm, –C*H_2_*–CH=CH– (PUFA acyl group); d. 1.65-1.51 ppm, –OCO–CH_2_–C*H*_2_– (PUFA acyl group); e. and f. (C*H_2_*)_n_ (Saturated and Oleic acyl groups, respectively). *discriminating metabolites from statistical analysis.
