# Supplementary material for: Traceability of “Tuscan PGI” Extra Virgin Olive Oils by 1H NMR Metabolic Profiles Collection and Analysis
Source: Metabolites. 2018 Sep 30;8(4):60. doi: 10.3390/metabo8040060 (PMC6316653; doi:10.3390/metabo8040060)
Supplement: Supplementary file 1 [file metabolites-08-00060-s001.zip › FigureS2.docx]

**<http://www.metaboanalyst.ca/faces/ModuleView.xhtml>**

**OPLS-DA (2+4+0)**

**1. pareto scaling method: R^2^X=0.83 R^2^Y=0.74, Q^2^=0.57**

**2. log trasformation: R^2^X=0.965 R^2^Y=0.552, Q^2^=0.261**

**3. pareto e log transformation: R^2^X=0.827 R^2^Y=0.738, Q^2^=0.567**

**1. PARETO scaling**


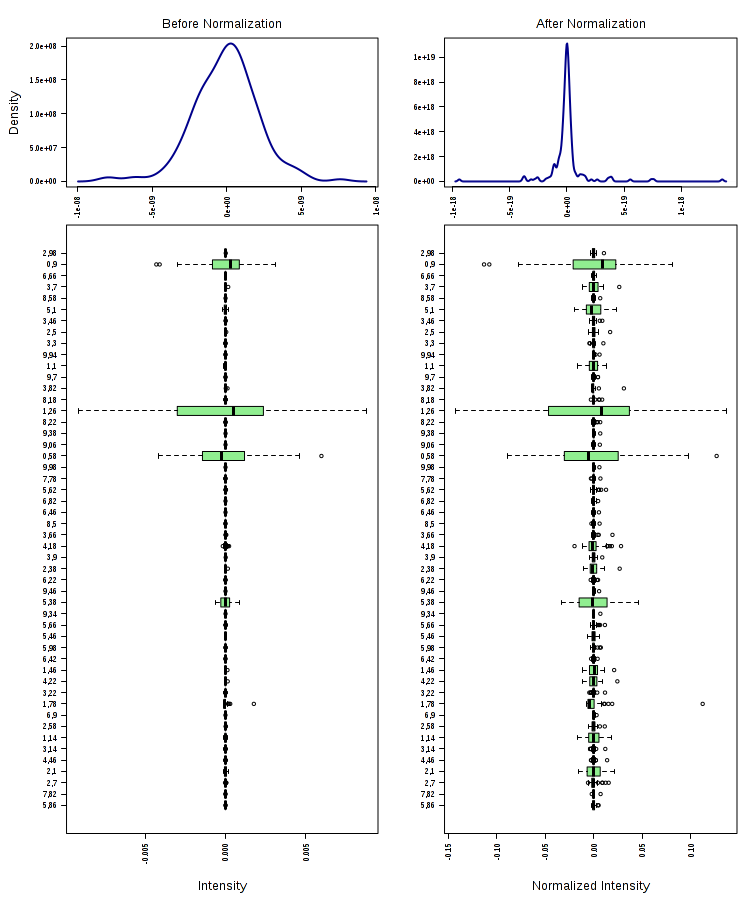


**2. Log transformation**


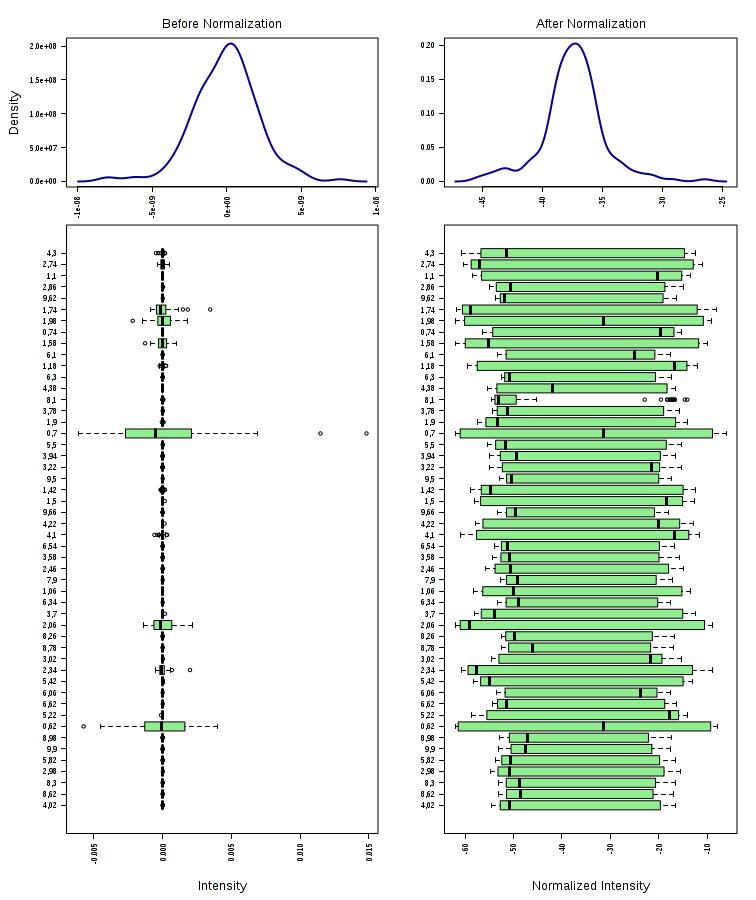


**3. log e pareto**

**
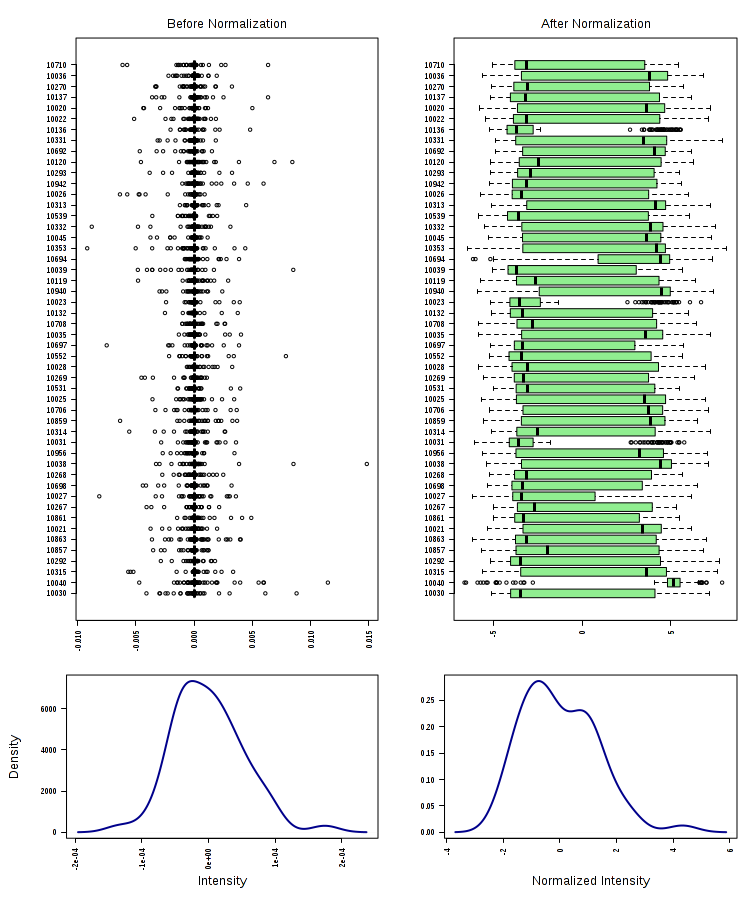
**
